# A 12-month projection to September 2022 of the COVID-19 epidemic in the UK using a dynamic causal model

**DOI:** 10.3389/fpubh.2022.999210

**Published:** 2022-11-15

**Authors:** Cam Bowie, Karl Friston

**Affiliations:** ^1^Retired, Axminster, United Kingdom; ^2^Wellcome Centre for Human Neuroimaging, University College London, London, United Kingdom

**Keywords:** Coronavirus, post-acute COVID-19 syndrome, incidence, vaccine effectiveness, compartmental models, public health methodology, epidemiology—descriptive

## Abstract

**Objectives:**

Predicting the future UK COVID-19 epidemic allows other countries to compare their epidemic with one unfolding without public health measures except a vaccine program.

**Methods:**

A Dynamic Causal Model was used to estimate key model parameters of the UK epidemic, such as vaccine effectiveness and increased transmissibility of Alpha and Delta variants, the effectiveness of the vaccine program roll-out and changes in contact rates. The model predicts the future trends in infections, long-COVID, hospital admissions and deaths.

**Results:**

Two-dose vaccination given to 66% of the UK population prevents transmission following infection by 44%, serious illness by 86% and death by 93%. Despite this, with no other public health measures used, cases will increase from 37 million to 61 million, hospital admissions from 536,000 to 684,000 and deaths from 136,000 to 142,000 over 12 months. A retrospective analysis (conducted after the original submission of this report) allowed a comparison of these predictions of morbidity and mortality with actual outcomes.

**Conclusion:**

Vaccination alone will not control the epidemic. Relaxation of mitigating public health measures carries several risks, which include overwhelming the health services, the creation of vaccine resistant variants and the economic cost of huge numbers of acute and chronic cases.

## Introduction

The recent abandonment of meaningful public health control measures in the UK in August 2021 provides a natural experiment, offering a base-line control population for other countries to assess the effects of their own public health interventions. It does so by providing a fecund opportunity for COVID-19 to spread throughout the population for months to come. How many people will be infected in the next year? How many will suffer long-COVID? How many more people will die of COVID-19? How effective are the vaccines used in the UK—BNT162b2 (38%) and ChAdOx1 (61%)? What will be the likely re-infection rate as vaccine-induced immunity wains?

Dynamic causal modeling is well-suited for predicting the effects of letting the virus sweep through the population because it combines conventional epidemiological models with behavioral modeling at the population level (i.e., it models and predicts both fluctuations in prevalence and contact rates) (1). Its predictions can provide a base-line for other countries as they monitor the effects of their public health control efforts. “If you do nothing except vaccinate—this is what will happen.”

## Methods

### Dynamic causal models

An advantage of dynamic causal models is that they are designed to continually assimilate data and update model parameters, such as transmissibility of the virus, changes in physical distancing and vaccine coverage—to accommodate changes in population dynamics and virus behavior. The September 25th, 2021 model was used to explore the effect of increased transmission risk of the Delta variant and the likely seasonal effect of the coming winter. Vaccine effectiveness with Delta and the curtailment of physical distancing as well as the potential benefit of a successful find, test, trace, isolate and support scheme were also incorporated into the model.

#### General features of dynamic causal models

The model is fully described—in accord with good practice in open science—and a weekly dashboard provides up-to-date estimates and projections (2). The annotated software is freely available and can be used to model datasets from other countries (3–7). Other researchers have used this kind of state space modeling (8–10), but not with the added features of the dynamic causal model used in our work, which includes a form of agent-based behavioral modeling. In other words, the conventional SEIR model is absorbed into a larger state space model that accounts for changes in behavior (e.g., tendency to self isolate) and testing (e.g., availability and uptake).

As such dynamic causal modeling stands apart from most modeling in epidemiology by predicting *mitigated* outcomes—and quantifying the uncertainty associated with those outcomes. This stands in contrast to quantitative epidemiological forecasts that do not consider the effect of prevalence on socio-behavioral responses. Usually, these projections are over few weeks—and rest upon fitting curves to the recent trajectory of various data; e.g., (11, 12). In contrast, dynamic causal modeling considers what is most likely to happen, based upon a generative model that best explains all the data available. This mandates a model of socio-behavioral responses that mitigate viral transmission, such as physical distancing, lockdown, testing and tracing. In turn, this requires a detailed consideration of how various sorts of data are generated. For example, it has to model fluctuations in testing capacity and sampling bias due to people self-selecting when symptomatic. The advantage of this kind of modeling is that any data generated by the model can be used to inform the model parameters that underwrite fluctuations in latent states, such as the prevalence of infection. Latent states refer to those states of the population that cannot be estimated directly and have to be inferred from observable data.

Dynamic causal modeling therefore focuses—not on worst-case scenarios but—on the most likely outcomes, given concurrent predictions of viral transmission, responses in terms of behavioral interventions and changes in the way that the epidemic is measured (e.g., confirmed cases, death rates, hospital admissions, and testing capacity). Crucially, dynamic causal modeling brings two things to the table. The first is the use of variational procedures to assess the quality of—or evidence for—any given model. This means that the model adapts to the available data; in the sense that the best model is taken to be the model with the greatest evidence, given the current data. As time goes on, the complexity of the model increases, in a way that is necessary to explain the data accurately. Technically, log evidence (a.k.a., marginal likelihood) is accuracy minus complexity—and both are a function of the data (13). This means there is an optimal model complexity or expressivity for any given timeseries data.

The second advantage of dynamic causal modeling is a proper incorporation of uncertainty in the estimation of conditional dependencies. In other words, it allows for the fact that uncertainty about one parameter affects uncertainty about another. This means dynamic causal models generally have a large number of parameters, such that the conditional uncertainty about all the parameters is handled together. This furnishes a model that is usually very expressive and may appear over-parameterized. However, by optimizing the prior probability density over the model parameters, one can optimize the complexity (c.f., the effective number of parameters), using Bayesian model selection (14–16). Note that the ability to pursue this form of structure learning rests on being able to estimate the model evidence or marginal likelihood, which is one of the primary *raisons d'être* for the variational procedures used in dynamic causal modeling (17–19).

These potential advantages can be leveraged to model a large variety of data types, to fit an expressive model of epidemiological trajectories and, implicitly, produce posterior predictive densities over measurable outcomes. In other words, parameterizing behavioral responses—such as physical distancing—as a function of latent states, enables the model to guess how we will respond in the future, with an appropriate uncertainty. This is the basis of the predictions of mitigated responses above.

#### Specific features of this dynamic causal model

The model includes all the standard SEIR (susceptible, exposed, infected, removed) features of the commonly used models of infectious disease but in addition incorporates the interactions between the population and COVID-19. For example, people are more likely to stay at home if the prevalence is high or if they have not been immunized. These dependencies are estimated and only retained if they improve the ability of the model to account for the data. Having optimized the model and model parameters, one can then proceed with scenario modeling to evaluate the effect of interventions such as the influence of an enhanced find, test, trace, isolate and support system on the epidemic.

Standard SEIR models depend on the choice of parameters, some of which are unknown empirically and must be guessed. Dynamic causal modeling is, by comparison, relatively assumption free. However, one must specify prior ranges for parameters (just like for SEIR models) but the dynamic causal model adjusts the parameters to fit the data in the most efficient and parsimonious way possible. Not only does the model provide estimates and projections of variables such as the death rate, the effective reproductive number, incidence, and prevalence but it also estimates of transmissibility, susceptibility, latent resistance, herd immunity, expected physical distancing behavior and vaccine effectiveness.

Two features provide insight into the way the model describes the interaction of the population and COVID-19. The first is the accuracy of the model in modeling the past stages of the epidemic. The second is the ability of the model to predict what will happen if we carry on as we have so far.

### Data sources and assumptions

The latest data from Public Health England and the COVID-19 Infection Survey of the Office of National Statistics (ONS) (20, 21) were used. It is assumed that mitigation efforts in schools will not take place, that lockdown will not be re-imposed, and that no new more virulent variant will arrive despite our porous borders and minimal travel restrictions. IHME provides estimates of national incidence (22). The trend in the use of non-pharmaceutical interventions by the UK government is measured using the Oxford Tracker stringency index (23).

The changing transmissibility of the virus—as new variants emerge—is included in the model (modeled with a set of temporal basis functions). For example, the Alpha variant was estimated to be about 50% more transmissible than the original variants and the delta variant was estimated to be about 50% more transmissible than the Alpha variant. These estimates are consistent with empirical estimates (24–27). The mix of vaccines used in the UK up to September 15th, 2021 was ChAdOx1 −53%, BNT162b2 −45% and mRNA1273 (Moderna)−3% (28). The NHS vaccine scheme by September 15th, 2021 had provided two doses to 66% of the population (29).

Three scenarios were explored. The first (NPI1) provides the projections with baseline parameters. The second scenario (NPI2) improves the find, test, trace, isolate and support system from 30 to 50% effectiveness. The third scenario (NPI3) increases the find, test, trace, isolate and support system to 80% effectiveness.

The latest Office of National Statistics infection survey dated September 5th, 2021 finds 831,000 people self-reporting post-acute COVID-19 syndrome (symptoms persist more than 12 weeks after presumed COVID-19 infection) (30).

### Software

**Figure 1:**
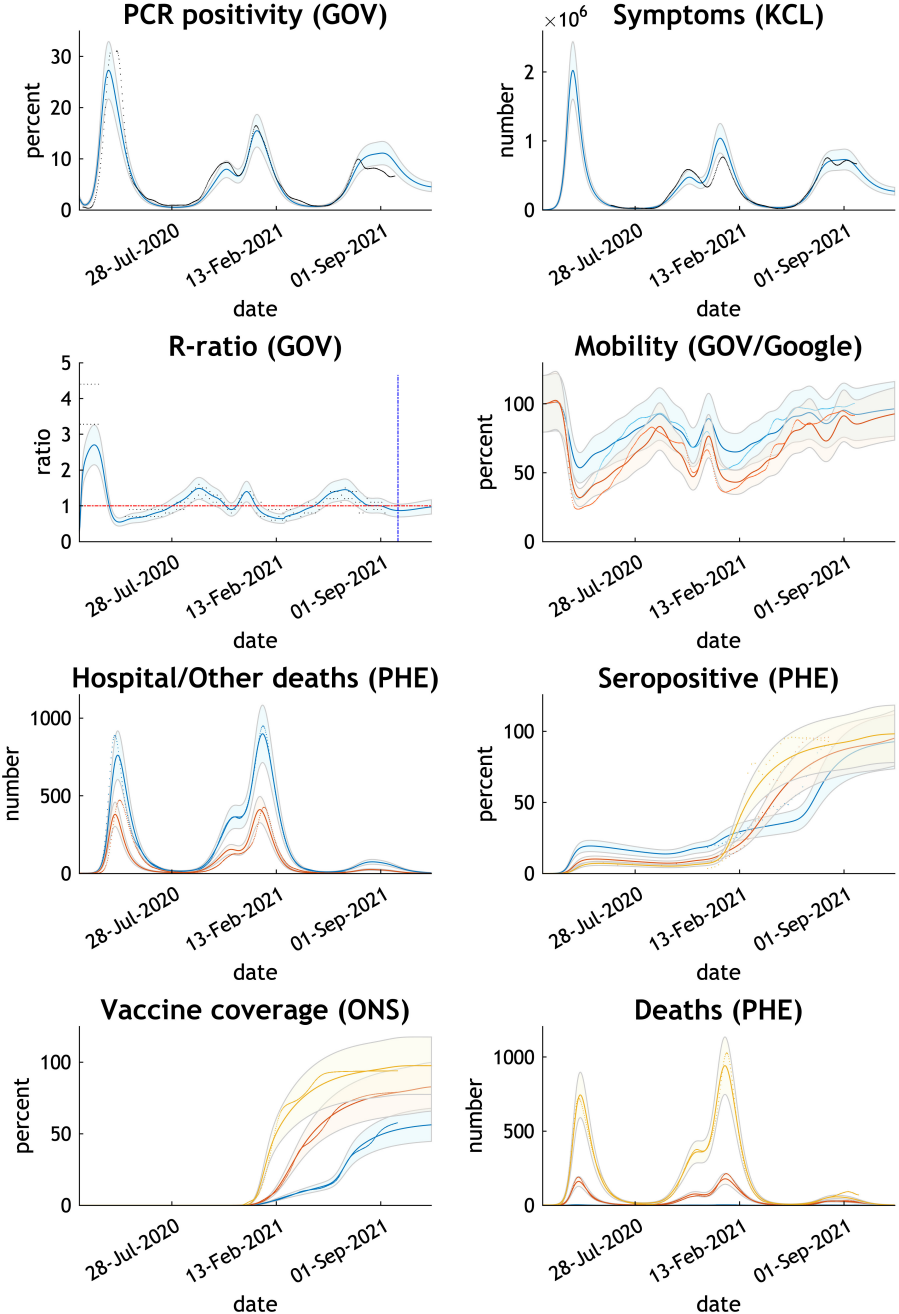
Comparing the actual with expected trends in eight measures of the COVID-19 epidemic UK—February 2020 to October 2021. Thick lines or dots are published data; thin lines and shades are Dynamic Causal Model estimates with 90% Bayesian credible intervals. GOV relates to NHS and Department of Health published data, KCL to Covid Health Study, PHE to Public Health England, and ONS to Office of National Statistics. Mobility GOV in blue; Google in orange. Hospital deaths in blue; other deaths in orange. Seropositive, vaccine coverage and deaths by age group - blue 15–34 years, orange 35–69 years, yellow 70+ years.

The figures in Figure 1 can be reproduced using annotated (MATLAB/Octave) code that is available as part of the free and open-source academic software SPM. The routines are called by a demonstration script that can be invoked by DEM_COVID, DEM_COVID_X, DEM_COVID_T, DEM_COVID_I, or DEM_COVID_LTLA at the MATLAB prompt. At the time of writing, these routines are available in the development version of the next SPM release. An archive of the relevant source code for each publication is available from figshare.

The remaining results in this paper can be reproduced using modified scripts found at https://www.dropbox.com/sh/79f9xu7dkd4kjul/AACb41iy4pjgPQlnXWG_VIZAa?dl=0.

The routine data used in the manuscripts are available from the COVID-19 Data Repository by the Center for Systems Science and Engineering at Johns Hopkins University, Coronavirus (COVID-19) UK Historical Data by Tom White and GOV.UK Coronavirus (COVID-19) in the UK. The CSV files must be available from the MATLAB path. The specific data on vaccine effectiveness are found in the Office of National Statistics and Public Health England publications (31, 32).

## Results

### The UK epidemic curve from February 2020 to September 2021

The chosen parameters adjusted by the model reproduce the epidemic curve and infection sequelae experienced by the UK up to the time of writing (Figure 1). The model estimates antibody immunity induced by COVID-19 infection and/or vaccine is lost in 284 days.

### Model predictions up to September 2022

**Figure 2:**
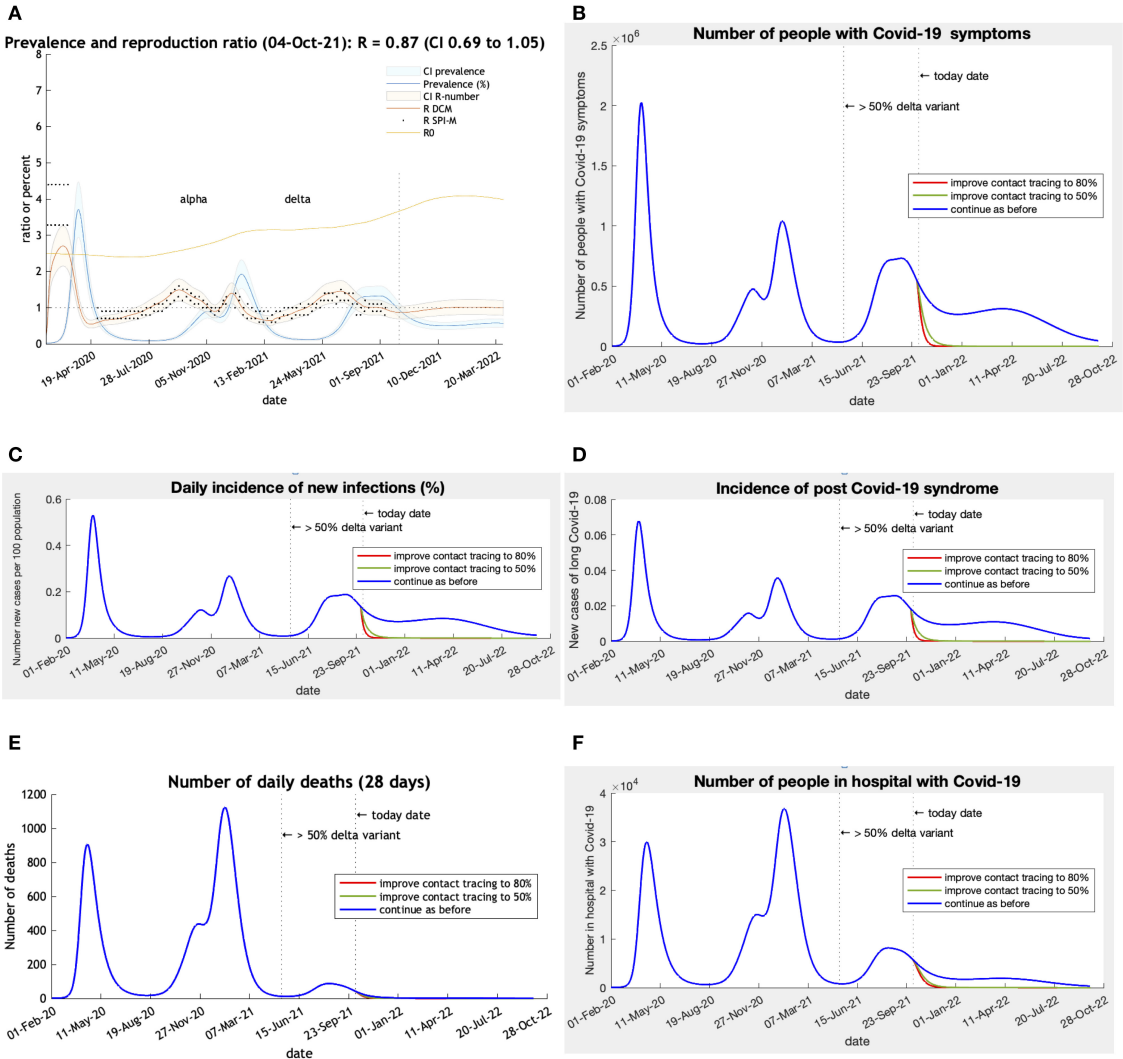
**(A–F)** CI is 90% Bayesian credible intervals. R DCM is the reproductive number calculated by the Dynamic Causal Model. R SPI-M is the published estimates of the UK government SPI-M-O committee. R0 is an estimate of the changing underlying transmissibility of the viruses in circulation. This estimate includes seasonality effects and may rise to about 6 as winter approaches. 50% Delta variant = date when Delta became the dominant variant. Scenarios: Blue—base-line predictions; Green—Find, test, trace, isolate, Support improved from 27% to 50% effective on 1st October 2021; Red—improved from 30% to 80% effective (80% close contacts isolate within 3 days) on 1st October 2021. Post-Acute Covid-19 Syndrome (using Office of National Statistics definition) assumed to occur in 3.36% of incident cases (19).

The projections illustrate the depth and persistence of the future epidemic in the UK in terms of morbidity and mortality, transmission characteristics, testing capacity, hospital utilization and disruption due to acute and chronic symptoms over the next 12 months if the government continues to withhold public health infection control measures (Figure 2).

### Vaccine effectiveness

The response in the UK to a prolonged wave of COVID-19 infections into the summer of 2022 is moderated by the high vaccine coverage. The vaccines reduce transmission by half compared to the original variants in circulation and pathogenicity for serious illness by 86% and deaths by 93% (Table 1). The effect for an individual is that two-dose vaccination reduces the risk of infection from 100 to 37%, and death from 100 to 0.3%.

**Table 1:** Vaccine effectiveness against COVID-19 Delta variant estimated by the dynamic causal model in UK in August 2021.

**Vaccine effectiveness with respect to Delta variant—** **September 2021—UK**	**Parameter derived from published literature**	**Estimated by DCM^a^ model**
Preventing exposure to infection:	75%	63.2% (CI^b^ 61.5–64.9)
Preventing transmission following infection:	72%	44.2% (CI 40.9–47.4)
Preventing serious illness when symptomatic (age 15–34):	76%	85.5% (CI 85.0–85.9)
Preventing serious illness when symptomatic (age 35–70):		85.6% (CI 85.2–86.1)
Preventing fatality when seriously ill:	91%	93.3% (CI 92.8–93.7)
**The reduction in risk from 100% after two doses of vaccine**		
Relative risk of infection		36.8%
Relative risk of mild illness		32.8%
Relative risk of severe illness		4.7%
Relative risk of fatality		0.3%

a Dynamic causal model.

b 90% Bayesian credible intervals.

### The long-term consequences

The trends in morbidity illustrate the consequences of allowing the epidemic to run in an uncontrolled manner through the community in the UK. The model can calculate its cumulative effect on case numbers, deaths, tests and hospital admissions (Table 2). Tests double, cases increase by two-thirds, hospital admissions by a quarter and deaths by 5% over the coming 12 months. An effective find, test, trace, isolate and support system in conjunction with the vaccine program would more or less stop further cases, hospital admissions and deaths.

**Table 2:** The cumulative effect of uncontrolled spread of COVID-19 in the UK—from February 1st, 2020 to October 1st, 2021 and to October 1st, 2022.

**Cumulative totals since February 1st, 2020**	**October 1st, 2021**	**October 1st, 2022**
**Scenario assuming FTTIS system^a^ is 25% effective**
Estimated incidence	37,124,370	60,697,287
Confirmed cases by PCR^b^	7,643,136	16,002,831
Deaths within 28 days of a positive PCR test	136,207	142,437
Tests (both PCR and LFD^c^)	302,707,235	636,316,101
Hospital admissions	536,258	684,004
Post-acute COVID-19 Syndrome^d^	831,000	1,358,661
**Scenario assuming FTTIS system improves to 80% effective on October 1st, 2021**
Estimated incidence	37,124,370	37,711,451
Confirmed cases by PCR	7,643,136	7,871,363
Deaths within 28 days of a positive PCR test	136,207	137,216
Tests (both PCR and LFD)	302,707,235	627,227,250
Hospital admissions	536,258	548,438
Post-acute COVID-19 syndrome	831,000	840,965

a Find test trace isolate and support.

b Polymerase chain reaction test.

c Lateral flow device test.

d Post-acute COVID-19 Syndrome definition—self reported symptoms more than 12 weeks after presumed COVID-19 infection used by Office of National Statistics (33).

### Retrospective analysis: Comparing the predicted and actual outcomes for October 1st, 2022

The Dynamic Causal Model projects the past into the future assuming conditions such as increased transmission risk, and the levels of testing and non-pharmaceutical interventions remain as before. Differences between the projected and actual numbers should be able to be explained by the features of the epidemic that have changed over the 12 months (Table 3). Since October last year the use of non-pharmaceutical interventions have been abandoned as measured by the Oxford stringency index falling from 41.2 in October 2021 to 11.1 in October 2022 (23, 34). The Omicron variant was designated a variant of Concern on November 26th, 2021 and became the dominant variant in the UK on December 13th, 2021 (35).

**Table 3:** Predicted and actual cumulative totals of incidence, deaths, tests, hospital admissions, and long COVID between February 1st, 2020 and October 1st, 2022 in the UK.

**Cumulative totals from February 1st, 2020 to**	**October 1st, 2021**	**October 1st, 2022**	**October 1st, 2022**	**Data source of actual numbers for October 1st, 2021 and 2022**
**Scenario assuming FTTIS is 25% effective**	**Actual**	**Projected in October 2021**	**Actual**	
Estimated incidence	15,153,730	60,697,287	105,678,303	IHME
Confirmed cases by PCR	7,643,136	16,002,831	22,241,311	UK Government COVID-19 dashboard
Deaths within 28 days of a positive PCR test	136,207	142,437	177,977	UK Government COVID-19 dashboard
Tests (both PCR and LFD)	302,707,235	636,316,101	514,605,757	UK Government COVID-19 dashboard
Hospital admissions	536,258	684,004	993,657	UK Government COVID-19 dashboard
Post-acute COVID-19 syndrome	831,000	1,358,661	1,825,000	ONS Infection survey

Post-acute COVID-19 Syndrome definition—self reported symptoms more than 12 weeks after presumed C-19 infection—ONS Infection Survey September 5th, 2021 and September 3rd, 2022.

FTTIS = Find, Test, Trace, Isolate, and Support system which in between February 2020 and October 2021 was estimated to be 25% efficient; IHME, Institute of Health Metrics and Evaluation; PCR, polymerase chain reaction test; LFD, lateral flow device test.

#### Cases

The projected total number of new COVID-19 cases was underestimated by 43%. This is likely to be due to the arrival of the Omicron variants and the removal of all public health mandates such as mask wearing and other interventions in the UK. A functioning Find, Test, Trace, Isolate and Support system was never fully established (36). Routine contact tracing ended on February 24th, 2022. In-person testing at test sites and free lateral flow device tests became no longer available on April 1st, 2022. While it was predicted that 26% of cases would be confirmed by a PCR test, in the event only 21% were so confirmed. This was due to policy change increasing reliance on stand-alone LFD tests.

#### Deaths and hospital admissions

Deaths were underestimated by 20% due to the very high number of cases despite a falling case fatality rate. The number of tests carried out was overestimated by 24% due to the discontinuation of free LFD tests. Hospital admissions were underestimated by 31% which is higher than the underestimation of deaths as the vaccines used in the UK protect more people from deaths than non-fatal acute respiratory distress syndrome requiring hospital admission.

#### Long COVID

Long COVID was underestimated by 21%. ONS estimated 1.29% (831,000 on September 5th, 2021) and 2.82% (1,825,000 on September 3rd, 2022) of the population had self-reported post-acute COVID-19 syndrome of more than 12 weeks duration (33). This is equivalent to 2.2% of cases in October 2021, which if staying at that proportion of cases would increase to 1,359,000 by October 2022. In fact, the latest estimate from the national COVID-19 infection survey is 1,825,000 or 1.7% of infections. So, despite a lower proportion of infections developing long term symptoms, our underestimate can be explained by the higher number of infections which actually occurred.

## Discussion

The UK provides a baseline of public health inactivity which can be used to compare public health controls chosen by other countries. The vaccines used in the UK seem to be extremely effective at reducing morbidity and mortality although not so effective at reducing transmission. Loss of immunity in 284 days as estimated by the model is similar to the results (extrapolated to 344 days) of the Office of National Statistics study (37) (Supplementary Table S4) suggests booster doses of vaccine may be required.

Despite these very effective vaccines, the UK can expect a large further wave of COVID-19 infections resulting in over half a million additional post-acute COVID-19 Syndrome cases, 150,000 hospital admissions and 300 million additional tests. If other mitigating public health measures were employed to support the vaccine effects this further epidemic would be eliminated. Simply making contact tracing effective would achieve that possibility.

The size of the projected wave of infections provides fertile ground for new variants and the absence of border controls will allow new variants from other countries to invade the UK. A health service already exhausted will be rapidly overwhelmed. The lack of effective public health resources will be unable to respond to current or future variants. This means that the UK government may find it has to re-introduce restrictive lockdowns. The excellent scientific institutions in the UK will be able to monitor such features as genomic sequencing, vaccine efficacy, and new vaccine trials. How long the population is prepared to be the guinea pig remains to be seen.

One of the values of using dynamic causal modeling is the ability to test pre-conceived ideas and allow the epidemic itself to inform its own modeling as it unfolds. The method also offers a factorial view of the epidemic rather than a unidimensional view as provided by standard SEIR models. The dynamic causal model allows an interplay between the various effects of behavior, epidemiology and seasonality that are key to the control of the epidemic. For instance, the non-mandatory response to an increase in COVID-19 prevalence is one of the factors used in the model. This provides insight into how individuals will respond to surges in prevalence based upon responses to previous fluctuations.

The Dynamic Causal Model can project the past into the future assuming systematic changes in the infectivity of the virus, and the levels of testing and non-pharmaceutic interventions. Differences between the projected and actual numbers should, in principle, be explained by the features of the epidemic that have changed over the 12 months. Our predictions underestimated the size of the epidemic over a 12-month period. A retrospective analysis suggests that this underestimate is due to the emergence of the Omicron variants and the abandonment of non-pharmaceutical interventions by the government.

The limitations of dynamic causal modeling predictions are non-trivial. While the model is able to factor in past behavior it cannot predict the biological characteristics of a new COVID-19 variant. However, the characteristics of new variants such as increases in transmissibility, decreases in virulence, and their response to vaccines can be included in the model—and validated against empirical estimates. Finally, it should always be acknowledged that uncertainty about the model *per se* is often ignored in this kind of modeling. In other words, although the model has been updated to maximize model evidence, it is just one model and other functional forms may, in principle, have a greater evidence and, implicitly, predictive validity.

In summary, the lessons for other countries are clear. Do not depend solely on vaccination. Relaxation of mitigating public health measures carries several risks—overwhelming the health service again, the creation of vaccine resistant variants, and the economic cost of huge numbers of acute and chronic cases.

## Data availability statement

The original contributions presented in the study are included in the article/supplementary material, further inquiries can be directed to the corresponding author.

## Author contributions

CB chose the topic, wrote the scripts for the scenarios and modifications to the DCM model, and wrote the draft paper. KF designed the DCM model. Both authors wrote the final version of the paper, contributed to the article, and approved the submitted version.

## Funding

KF was supported by Wellcome through core funding to the Wellcome Center for Human Neuroimaging, UCL Queen Square Institute of Neurology (203147).

## Conflict of interest

The authors declare that the research was conducted in the absence of any commercial or financial relationships that could be construed as a potential conflict of interest.

## Publisher's note

All claims expressed in this article are solely those of the authors and do not necessarily represent those of their affiliated organizations, or those of the publisher, the editors and the reviewers. Any product that may be evaluated in this article, or claim that may be made by its manufacturer, is not guaranteed or endorsed by the publisher.
